# The Prevailing Epidemic in the Northwest

**Published:** 1863-03

**Authors:** 


### EDITORIAL AND MISCELLANEOUS.

### THE PREVAILING EPIDEMIC IN THE NORTH-WEST.

MESSRS. EDITORS :—An epidemic has been and is still, to some extent, prevailing in this vicinity, that I should like very much to have your opinion on, and suggestions in regard to, either by letter or through the columns of your "Journal." I diagnosed it, Cerebro-Spinal Meningitis, and I had able counsel who concurred. But that you may judge for yourself, I give you the symptoms. The disease is ushered in with a chill, or shivering, which, the patients say, is not like an ague chill, but a shivering without much sense of coldness. The chill is preceded by intense pains in the head, back of the neck, and in various parts of the body, but occasionally in one of the extremities only. The pain and aching continues with greater or less severity throughout the course of the disease. The chill in some instances lasts twelve hours, but in most not over three or four. Violent vomiting soon sets in, and continues for about twenty-four hours ; provided, the patient survives so long. Constipation very obstinate. In some cases, it is impossible to get an evacuation per rectum by cathartics introduced into the stomach. Extremities cold as clay, and very difficult to warm for a day or two. Pulse weak, but not much accelerated. Tongue little, if at all coated, but of a peculiar purple or livid color, at first, but in the course of the disease becomes covered with an "ashy" colored coat. In twenty-four or forty-eight hours, sometimes less, an eruption appears ; on the face, looking like measles, but on other parts of the body the spots are much larger and extremely sensitive, the slightest touch often causing the patient to awake from a sound sleep with a cry of pain. In a few days the patient becomes sore all over, and the joints so stiff, that it is with great pain that he can be moved at all. In some cases, the spots, at first, are of a dark purple, almost black color, in others scarlet, but soon turn dark; these are the most favorable cases for recovery. The pupils are dilated, the left one most, and generally the right side is totally or partially paralyzed—some patients become deaf, dumb, or blind, or all three together, and one, though convalescent for over two weeks, cannot yet speak. Most of those attacked are delirious, but the delirium is not active. Out of thirteen cases treated by myself five died, and another died without treatment. There have been no new cases for more than a week. Death occurred in less than twelve hours in three of the cases. The treatment was actively stimulant and tonic, irritants to the spine, in two cases blisters—and bathing with hot brandy and capsicum. The result was not very flattering, but my experience has brought me to the conclusion, that the only hope of saving life, as the disease occurred here, is prompt, active and powerful stimulation, followed with tonics. The locality in which the disease occurred is on rolling prairie. With one or two exceptions, those attacked did not have chills last fall, so that lingering intermittents could not have been the cause of the attack.

There have been more rain and mud here this winter than for many years. The consequent dampness of the atmosphere may have been an exciting cause.

Yours respectfully,

Boswell Ward.

---

This is one of a large number of letters received on the same subject during the last season. The accounts given by correspondents are very similar, although the incursions of the disease have been observed over not less than five States of the North west.

Of it, very little is found in the text books—nevertheless, it may be said to be well known to many in the profession. Well known to the extent at least of diagnosis—unfortunately, not yet very well known so far as reliable therapeutics are concerned.

It occurs both in the sporadic and epidemic forms. Of the former, several well marked cases have been brought to our notice in this city quite recently; of the latter, many of the interior towns have afforded sad evidences.

Although having the termination *itis* to its technical appellation, it is far from being an inflammatory affection, in the ordinary acceptation of the phrase. There is a modification of the nutrition in the cerebro-spinal substance, with often resulting destruction of the tissues, but the changes are more like those ensuing in typh diseases or cholera than active inflammation.

In observing the phenomena presented, we can scarcely avoid the belief that they are caused by some specific *materies morbi*, and yet it is pretty clear that this must have been generated within each system by coincidence of external causes operating upon it. The causes operating without—the poison generated within. Particular stages of the disease present phenomena strikingly like those resulting from uræmic poisoning.

According to our own observation, it is more likely to occur in winters with a variable temperature—where a few days of intense cold are rapidly followed by days of thaw, mud and rain. Neither uniformly cold nor warm weather are so likely to engender it.

It does not seem to select those previously debilitated by disease in preference to the robust. It does not select any particular age or sex, neither are any exempt. Nor does it seem to be influenced materially by localities—those upon the highlands and those upon the lowlands—the rich and the poor are quite impartially seized.

The methods of attack are various, and yet all coincide in representing a potent influence upon the nervous system. More commonly, this is manifested by distinct *rigor* gradually passing to general coldness, absence of pulse in the extremities and coma, very like that of pernicious remittent; and this without any marked local symptoms.

At other times, *pain* is the prominent symptom—pain more frequently in the occipital or nuchal region, but very often in other parts of the head, in the shoulders, trunk, hips or extremities. This pain is usually sharply circumscribed within a small compass, but may extend. It becomes more and more intense until the patient grows delirious, apparently from its severity, and then ultimates in coma, after which the disease takes the same course as when ushered in by the chill. Or it may speedily be replaced by the rigor.

The *suddenness of the attack* and rapid progress to grave symptoms, are very characteristic.

The delirium which ensues upon the pain, or which supervenes upon the coma, is of the most violent character, incessant jactitation, and often vociferous and incoherent cries.

Sometimes at the very outset, but usually not until the lapse of several hours, from three to twenty-four, there occurs a peculiar discoloration or eruption upon the surface, but exceedingly variable in its form. Sometimes it consists of a simple petechial point, and again of a dark discoloration of a large surface like ecchymosis, or rather the subcutaneous extravasation of purpura. More frequently there is no elevation of the cutis, but occasionally there is an elevation of the epidermis, like bullæ, with thin, dark sanious contents.

We have never seen it look like the eruption of measles, as suggested by our correspondent, but very often somewhat like the mulberry rash of continued fever.

The form of the eruption is not diagnostic. Mainly, the evidences presented by it are those of a diffluent, disintegrated state of the blood. The prognosis is modified by the brightness or lividity of the hue of the discoloration.

The eruption, or discoloration, is not always present, but so frequently as to well entitle the disease to its popular name, "spotted fever."

Among the most remarkable symptoms may be mentioned the persistent tonic contraction of the muscles of the back of the neck, sometimes deepening into opisthotonos; convulsions; paralysis, particularly of the optic and auditory nerves; remarkable soreness of the surface and joints to touch or motion.

There may be intolerance of light and sound, but oftener the special nerves involved are paralyzed. While there is the most excruciating hyperæsthesia of the general surface, the finger may be placed on the cornea with no flinching by the patient.

The vocal muscles may be paralyzed, as also those of deglutition, but these forms are more rare. Hemiplegia is not infrequent; or the paralysis may be confined to a single extremity. Strabismus, unequal size of the pupils, twitching of the facial muscles, &c., &c., are incidental symptoms.

The pulse, at the outset, is small, thready, tremulous, irregular, soft, and speedily lost at the wrist. On the occurrence of reaction or remission it increases in fullness and force. About always during the primary stage it is slower, but during reaction it may run up to 120 or 150.

The skin in most cases is dry as well as cold, but in perhaps 25 per cent. of the cases it is moist. In all cases it is quite variable in this respect at different hours of the day.

The tongue is more or less enlarged and flabby, with indentations of the teeth upon its edges. It is pale and moist, with a coating of a pale ash or white color passing to yellow and brown, but this is not distinctive.

Nausea and vomiting are occasional, but not constant symptoms, the matters thrown up being the normal contents of the stomach, or, in bad cases, decomposed blood, bile, mucus, &c.

The bowels are variable. Some cases are natural, in others there is obstinate constipation, and in others still, severe diarrhœa with discharges of grumous and offensive matters.

The respiration is ordinarily laborious and irregular, sighing, and perhaps stertorous, more frequent than normal, while consciousness continues, but becoming slower with advancing coma.

The urine is generally scanty, depositing a plentiful sediment intermixed with ropy mucus, and disorganized blood corpuscles. But in some instances, we have known the urine abundant and of low specific gravity.

The first stage may terminate in death in a few hours or, after an interval of from six to twenty-fours, then may occur a marked remission of the symptoms—a very deceptive remission, leading both physician and patient to believe the difficulty pretty much over. Supervening upon the remission rapidly, or it may be upon a gradual awakening from the comatose state, there will be intense febrile excitement, great heat of the surface, rapidity and strong tension of the pulse, arterial throbbings, flushed face, turgidity of the conjunctiva, with extreme thirst, &c. This is liable to be attended with the highest grade of delirium and frantic muscular jactitation, requiring several attendants, perhaps, to keep the patient in his bed.

The delirium may, after a while, merge in coma, apparently from utter exhaustion; or it may gradually moderate in violence and pass into the low muttering character, with subsultus, &c., the patient being laid upon his back with the extremities fixed and painful to touch and motion; or in some cases turned rather to the prone position, with, in either case, the posterior muscles of the neck fixed rigidly, drawing the head strongly backwards. Occasionally it is drawn laterally backwards, but is always in a state of rigid immobility.

In this stage the bowels are usually inactive and the urine either scanty or wholly retained. The skin is hot, but not infrequently reeking with a peculiarly offensive perspiration. Irregular or partial sweats are very common. Convulsions, paralysis, pain, and other evidences of central nervous disorder, are largely aggravated.

The patient may die in this stage, or the disease may pass on with irregular febrile phenomena into the low, lingering typhoid stage, stretching over even forty or sixty days before the establishment of a tedious convalescence. The low fever which so often supervenes upon severe attacks of Asiatic Cholera much resembles it.

From the outset there will be presented intermingled symptoms, according to the involvement of particular organs in secondary lesion. The lungs are especially liable to severe or even fatal disorder, so much so indeed, as by the gravity of its manifestations almost entirely to mask the original disease.

Dr. Condie, in a note to the American edition of Watson's Practice, includes a description of this form under the head Typhoid Pneumonia, but it is unnecessary to say that the disease described is but the one now under notice with secondary complication. Ulceration of the throat, the writer has observed as a complication in several cases—the destruction of tissue proceeding of itself to an alarming extent.

Above all, Erysipelas is the most dangerous of complications. It is easily kindled upon the slightest abrasion of the surface, and often, without such a nidus, attacks the face and neck, extending to the scalp, or more rarely seizing the extremities. This erysipelatous affection is apt to be exceedingly destructive to the part attacked, giving rise to foul, slough ing sores, rapidly wearing out the patient by exhaustion.

Some of our friends who have observed this frequent concurrence of erysipelas, have been so struck by it as to believe the disease essentially an erysipelatous inflammation of the cerebro-spinal tissues, as also of those organs involved secondarily.

The abdominal viscera sometimes experience the full force of the morbificinfluence, but in greater part are comparatively little disturbed.

Epistaxis, hæmatorrhœa, hæmatemesis, and hæmaturia are occasionally present to a dangerous extent.

Among the sequela we note, deafness, blindness, partial paralysis of motion, tuberculosis, albuminuria.

Marasmus in both children and adults, and phthisis in the latter are especially common. The constitution seems broken down, and perhaps years elapse before the patient recovers his original vigor.

With regard to *post mortem* appearances there has been much apparent discrepancy of description. But by attention to one single fact this can readily be explained, and that is, the stage of the disease at which death occurred. It is also necessary to separate incidental complications from the primary affection.

If the patient die in the first stage within a few hours from the attack there may be very few signs of local change. The blood will be found settling to dependent portions, and with a loose, gory and diffluent clot. There will be found stasis of blood in the capillaries of the cerebro-spinal meninges, particularly the pia mater and dura mater, rarely of the arachnoid. The superficial vessels of the convolutions will be distended, and those upon the walls of the ventricles likewise full, presenting here and there spots like ecchymosis, though more florid in hue. Sections of the cerebral substance will show a similar condition of the bloodvessels, as manifested by the large red points on cutting across them. The medulla oblongata and spinal meninges and cord at the upper part will exhibit similar appearances. The stasis of semi-dissolved blood is so considerable that the grey and white tissues gain a pinkish tinge.

More or less circumscribed softening is found, usually involving the cortical substance of the cerebrum, the inferior surface of the cerebellum, and the floor of the lateral ventricle. In fine, a lessening of the firmness and density of the whole mass is evident on careful inspection. Effusion of discolored serum into the cavities is present now and then to a large amount.

If the case has run on to high reaction, all the results of inflammation may be present. In this case, the arachnoid is apt to be thickened and with loss of its transparency. In the exuded matters are found pus and lymph corpuscles, or the exuded lymph forms a quasi membrane, loose, creamy, and readily broken down, upon the surface or along the course of the large vessels. The greatest quantity is at the base of the brain, and always at the optic commissure. It is also stated to be found on the corpora quadrigemina, the medulla oblongata and around the third pair of nerves where they penetrate the arachnoid membrane. Wherever the exudation takes place it is likely to be stained by the dissolved hæmatin.

In the spinal region, the morbid changes affect mainly the meninges, the substance showing little modification of structure, the most observable change being softening. The exudation, discoloration and softening are most observable about the roots of the cervical nerves.

The thoracic and abdominal lesions present nothing distinctive. Where the lungs become involved there are the ordiary evidences of stasis of blood and effusion, or where the later stage is reached, the usual results of inflammation. The same remark may be made with regard to the intestinal tube.

It is proper to remark, that whilst in about every case there is on *post mortem* some visual evidence of local disease of the cerebro-spinal region, nevertheless, in very many this evidence is very slight. The disease is capable of killing the patient speedily or slowly, and yet leaving behind it no trace of its footsteps. But the only constant changes, when any are present, are in this region. Here, as elsewhere, the facts teach that the immediate molecular changes, upon which life and death depend, are beyond the sphere of either our aided or unaided observation. We necessarily infer their existence, precisely as we infer the reactions of a chemical mixture by observations of results and of things which we can see and handle, and weigh.

In common with many other potent influences which suddenly strike down the powers of life—the cause or causes of this terrible disease may produce at once necræmial symptoms, so profound, that particular manifestations of the special cause will not take place. We shall see that thus far the diagnosis is not of great importance. Under this condition the most dissimilar causes coincide in effect. Malignant typhus, small pox, scarlatina, rubeola, diphtheria, Asiatic cholera, erysipelas, pernicious remittent, uræmia, &c., &c., are severally capable of producing this condition.

In this case, an overwhelming cause acts especially upon the cerebro-spinal centre, or, secondarily, upon other organs. The blood from the beginning is poisoned and scarce living, and death may ensue from this poisoning of the blood, or later from the local changes in the brain and cord, or in other instances, from involvement of other vital organs. The only forms of disease with which it is liable to be confounded are pernicious remittent (congestive fever) and typhus.

Yet, be it observed, in the progress of both these forms, cerebro-spinal changes may occur which render the diagnosis impossible; and this need not be deemed unfortunate, because at the same time it is unnecessary. Since enlightened medical men have ceased to regard diseases as entities, and no longer rely upon a routine treatment, the diagnosis of names has yielded place to the search for causes and conditions; and treatment has been placed upon a broader and more certain basis.

The reader who has carefully noticed the peculiar disturbances of the nervous system and muscular apparatus portrayed in the communication of our correspondent, and further designated in our own remarks, cannot fail to see that they are exceedingly rare in the so-called congestive fever of "malarious" districts. There is no periodicity about it. It cannot be merely a severer form of that fever, for it occurs in districts, indiscriminately, where that fever is known and where it is not. It occurs at a season of the year when pernicious remittent notoriously loses its virulence, and, indeed, is scarcely ever present save in cases which have been especially liable to attacks of periodical disease and whose constitutions have been broken down by previous disease. Briefly, the paralysis of special sensation and voluntary motion; the hyperæsthesia of the surface and joints, or circumscribed localities; the opisthotonos and convulsions; the petechiæ and vibices; the absence of periodicity, notwithstanding remarkable remissions; locality and times of occurrence; the sequela; these and many other particulars distinguish the disease from "congestive fever" with no difficulty or uncertainty. The influence of Quina is wonderfully diagnostic. In pernicious remittent it is prompt, powerful and certain. It is *the* remedy, if all others were ignored. In the disease under review it is scarcely noticeable in effect, and wholly unreliable—nay—comparatively worthless as a remedy. The "congestive fever" bows beneath its potent influence at once—the "spotted fever" scorns its impression.

Practically, the *physiognomy* of the two diseases is as diverse as the features of strangers.

Nearly the same distinctions separate it from "maculated typhus." It is not a severer form of typh disease, because it is rarely developed under those known circumstances which develop the latter. The nervous manifestations at the outset are wholly diverse; the mode of the attack and progress of the case are dissimilar, save that in some cases a low fever follows the primary lesions; but well marked, and indeed some of the very severest, cases are arrested and convalescence occurs without any such secondary fever.

The eruption, or rather extravasation of dissolved blood beneath the skin, is wholly unlike the maculæ of typhus or the roseolar spots of typhoid. Such extravasations do not constitute the peculiar eruption of the typh disease.

Typh fevers are the creation of *ochlesis*, of bad ventilation, filth and improper food. This clearly depends on some unknown meteorological influence, entirely apart from the accumulation of human beings in crowded places; independent of decomposing organic matter; independent of confined air.

It attacks equally all ages, from the infant to the veteran—the out-door laborer and the lady in the parlor.

Presenting some symptoms in common with the typh diseases, its history and its *physiognomy* are as diverse from them as yellow fever is from bilious remittent. To confound them under one denomination, will render it necessary to re-write the history of typhus and make the term as comprehensive as the word *fever*.

It would be a happy circumstance if the treatment were as satisfactorily determined as the character of the disease. But, as before remarked, this is unhappily not the fact. But much can be done, and many lives saved which without it would certainly be lost.

The proportionate mortality in the early periods of this, as of other pestilential epidemics, is something frightful to contemplate; but there always remains this well established fact that, under about any respectable treatment, the fatality steadily diminishes with time, and the later cases will be quite controllable.

The quacks outside and inside the profession take advantage of this truth, and all ears are speedily stunned by the praises of particular remedies and methods which they go around braying about. "Specifics" for it are as plentiful as for cholera in every city, village and hamlet—but, alas, they fail miserably in the real trial.

Under our own observation has come treatment which ought to make all classes of created things, from angels to jackasses, weep.

We have seen men try to bring on a crisis—the *death-crisis* to which we alluded last month—by bloodletting. Others attempt the same thing by emetics and mercurial purges. Steaming is perhaps the most popular method of producing exhaustion of the feeble remaining powers of life. We knew one physician to give upwards of fifty grs. of morphine to a boy within a few hours to relieve his terrible pain and suffering. He observed to the writer that "the boy did not die of the disease!" Incalculable quantities of brandy and quinine have been poured into the stomachs of the comatose; and there is no measurement of the amount of turpentine which has been thrown up *per rectum*.

The whole surface has been parboiled, roasted, and cauterized—What have we not seen done? We have seen the patient wrapped in the skins of animals torn reeking from the still quivering flesh; and have known an ox disembowelled and a tender girl put in the steaming cavity. There is no limit to human credulity—there is no bound to human assumption. Popular follies are the legitimate offspring of the dogmatic wisdom of some previous age.

Satisfactory treatment remains yet to be discovered. There are no specific remedies as antidotes. Yet experience points to certain things which seem to have done something—perhaps, *post hoc ergo propter hoc* after all.

And first we rank external stimulants. Rubefacients and artificial heat. Whatever will powerfully stimulate the surface, especially along the spine and the extremities. This not from any vague idea of internal pressure to be overcome by "determining to the surface," but by promoting tissue change in the parts and thus awakening energy of the circulation, and immediately excretion of effete matters and formation of new blood. The rubefacient becomes thus a general stimulant. With capsicum and mustard, ammonia and terebinthinates, pungent oils and friction—the means are abundant enough, and the practitioner can take his choice. Direct heat is a more questionable remedy from its ultimate sedative impression. The hot water or vapor bath speedily exhaust—still, when the surface is dry and rigid, these may be employed for a short time only. The alcoholic vapor bath is often serviceable. A potent stimulant is the application of ice for a few moments and then the alternation of hot epithems—this alternation to be frequently repeated. The intense local pain so often present in the primary stage may occasionally be controlled by large doses of morphia internally, but the amount required is apt to be so excessive that the hypodermic injection at the point of suffering will be found of greater advantage. But from the puncture there is always danger of erysipelas. The same remark applies to vesicants—primarily of excellent service, secondarily there is danger of sloughing sores.

Internally stimulants, boldly, but with due regard to their ultimate effect.

Thus the alcoholic stimulant, of whatever sort, when acting at all is quite disposed to produce the sedative impression or anæsthetic effect—checking tissue metamorphosis, hence the disappointment so frequently experienced on their administration during the cold stage, or in necræmial cases. The alcohol should be given only in moderate amount and combined with capsicum, piperin, monarda, or similar aromatic stimulants The Ol. Monarda here, as in Asiatic cholera, will be found quite efficient. Or Ethereal preparations, especially the Chloric Ether, in full doses with Sp. Cardamon Comp., Carb. Ammon. and Capsic. in full doses, answers well in some cases.

In our experience, the Tincture of Cantharides in full doses, is perhaps the most valuable of all internal stimulants. From twenty to forty drops every hour, until reaction ensues.

Where there is any appearance of erysipelas, or where there are evidences of dissolution of the blood, as evidenced by the deep livid shade of the spots, or by passive hæmorrhages, the Muriated Tincture of Iron should be simultaneously administered. Coinciding with the general effect of the Cantharides, it seems to especially prevent further destruction of the blood and facilitate its repair. Besides, it lessens the tendency of the Cantharides to injure the kidneys and produce strangury. But the awakening of the nervous system and of the excretory energy as shown by this result is scarcely to be considered as other than a favorable omen. Of course, when this occurs, the Cantharides should be replaced by some other stimulant. From ten to twenty drops of the Tincture of Iron should accompany each dose of the Cantharides, and the mixture be largely diluted on exhibition.

If convulsions occur, or pain be dangerously intense, anæsthetics by inhalation may be employed, and we should very much like to try here, as we have not yet had an opportunity, the Protoxide of Nitrogen, or "laughing gas." Theoretically and by analogy of its use in other cases, we believe it would be found an invaluable remedy. Its energetic stimulating power would probably favor speedy reaction.

We have not noticed any especial advantage from Quinine in the first stage, although we have given it in doses varying from two grains to half a drachm every hour. The stimulating effect of the small dose is inappreciable; the sedative and diaphoretic effect of the large dose is objectionable. But when the deceptive remission occurs, we have fancied some benefit was gained by several full doses, at intervals of one or two hours—the sedative effect seemed to lessen the severity of the subsequent exacerbation, although we have found that a hundred grains thus given, would not prevent its access.

At this time, something appears to be gained by relieving the bowels by a moderately stimulating cathartic, particularly when the cold stage has left behind it decided engorgement of the portal system. By this means the exacerbation is evidently ameliorated, and the tendency to pass afterwards into a low form of fever sensibly lessened. We usually, for this purpose, combine calomel with gamboge, rhubarb and soap, which usually operates efficiently in six or eight hours. Beyond this, or some milder cathartic, we have rarely found it useful to employ purgation. The patient cannot be cured by attempts to turn him inside out with either emetics or cathartics.

If, during the stage of excitement which now follows, the urine becomes tolerably free and charged with its solid constituents, the patient may be reckoned safe, but if it is suppressed the case is one of extreme danger. The practitioner is apt to be deceived by the apparent excitement, and hence to resort to excessive antiphlogistic measures—but this is a dangerous error. Collapse impends almost momentarily, and it is necessary to be on guard.

Saline diuretics, very moderate doses of the usual sedatives, and perhaps anodynes, may be used, but bleeding, vomiting and purging, or large doses of sedatives are out of the question. Ablutions with cold water, or the tepid bath, or showering the head, we have found in this case, even more useful than in the similar stage of remittent.

When in doubt, our own experience teaches us to say, *stimulate* even here. Destructive changes will thus be prevented, the liability to collapse lessened, and the tendency to tedious low fever, with doubtful final result, abated. But better do nothing than to do wrong or mischief.

The subsequent treatment of the disease is sufficiently simple, being merely that of the low fever, modified by such local complications as happen to be present. We have not time to follow up the subject at present. Indeed, we had intended but a few words supplementary to our friend's communication, but have been insensibly led along to a length of remark wholly unexpected.

Will some of our correspondents, where the disease is, or has been prevailing, prepare a communication for our pages on this subject?

